# Multimodality molecular imaging of the alveolar-capillary barrier in lung disease using albumin based optical and PET tracers

**DOI:** 10.1186/s43556-020-00020-1

**Published:** 2020-12-20

**Authors:** Andrei Molotkov, Nikunj Bhatt, Mikhail Doubrovin, John Castrillon, Christopher Massa, Adam Gerber, Jeanine D’Armiento, Monica Goldklang, Akiva Mintz

**Affiliations:** 1 Department of Radiology, Columbia University Irving Medical Center, 722 W. 168th St., New York, NY 10032 USA; 2 Center for LAM and Rare Lung Diseases, Department of Anesthesiology, Columbia University Medical Center, 722 W. 168th St., New York, NY 10032 USA

**Keywords:** PET, ^68^Ga-albumin, ARDS, COVID

## Abstract

Inflammatory changes caused by viruses, bacteria, exposure to toxins, commonly used drugs and even surgical intervention have the potential of causing abnormal epithelial permeability, which is manifest as infiltrative processes on computed tomography (CT), including the widespread infiltrates seen in COVID-19 pneumonia and acute respiratory distress syndrome (ARDS). We utilized a previously published mouse model of ARDS, intranasal delivery of LPS, to induce the alveolar-capillary barrier permeability seen in lung disease. We intravenously injected mice with Cy7 or 68-Gallium (^68^Ga) labeled mouse albumin and imaged using optical imaging (OI)/CT and PET. We observed significantly increased lung levels of Cy7-albumin on 3D OI/CT, which matched the abnormal appearance on microCT. This uptake correlated with fluorescence seen on sectioned lungs. To examine the translational potential of these findings, we radiolabeled albumin with ^68^Ga. We found that in mice with LPS-induced lung injury, ^68^Ga-albumin PET correlated with our optical imaging findings and demonstrated abnormal activity in the lung fields, indicative of abnormal epithelial permeability. These findings indicate ^68^Ga-albumin can be utilized as a sensitive translational radiotracer for quantifying the abnormal epithelial permeability that is seen in various lung pathologies, including COVID-19 induced pneumonia and ARDS. The ability to use Cy7-albumin 3D OI/CT imaging as a preclinical translational surrogate for ^68^Ga-albumin offers an accessible high throughput means to rapidly screen potential therapeutics against lung diseases that clinically manifest with endothelial permeability.

## Introduction

Under normal circumstances, the alveolar-capillary membrane prevents fluid from entering the alveoli and hindering gas exchange. The alveolar-capillary membrane (or barrier) is composed of surface epithelial cells of the alveolar wall (type 1 pneumocytes), the endothelial cells of the capillaries and the basement membrane between them. Various diseases result in increased permeability in the lungs that results in fluid filling the alveoli and preventing gas exchange. Acute lung injury (ALI) and acute respiratory distressed syndrome (ARDS) are caused by overproduction of cytokines, increased vascular permeability, protein-rich edema, and are leading causes of mortality in the patients [1]. Other causes of increased alveolar-capillary permeability in the lung include Pneumonitis and Pneumonia. Pneumonitis is an inflammation of lung parenchyma that is caused by a variety of airborne irritants. Pneumonia is pneumonitis triggered by bacterial, viral or fungal infections. Viral pneumonia with associated ARDS has emerged as a significant public health threat during the COVID-19 epidemic, with a significant minority of diagnosed patients suffering from lung infection and hospitalization [2]. Many of these patients end up requiring ventilators to achieve proper oxygenation. Of the patients placed on ventilators, ~ 60% succumb to the virus [3]. While many patients who suffer COVID-related lung disease have comorbidities, it remains unpredictable which patients will suffer from lung complications until they demonstrate compromised oxygenation and abnormalities on plain chest radiography or CT. In addition to viral pneumonia, bacterial pneumonia [4] is one of the leading causes of hospitalization and morbidity worldwide with a reported age-standardized death rate of 41.7/100000 [5]. Hospital-acquired pneumonia significantly complicates treatment and negatively affects clinical prognosis and patient recovery. Computer tomography (CT) and plain chest radiography are the most often used standard method of diagnosing increased vascular permeability in the lungs, which manifests as lung infiltrates [6]. However, the presence of infiltrates indicates the late effect of the disease process, which is significant fluid in the lungs, and may not be a real-time measure of triaging early disease, quantifying the dynamics of the underlying alveolar and airway epithelial permeability, or an early predictor of response to treatment.

Lipopolysaccharide (LPS) induced lung injury is a commonly used model for ARDS, mimicking the inflammatory response observed in ARDS patients [7]. Local tracheal inhalation of LPS triggers robust migration of neutrophils into the lung parenchyma by 72 h after the exposure [8] followed by secondary fibrosis. Molecular mechanisms of the cellular response to LPS involve LPS binding to toll-like receptor 4 (TLR-4) followed by activation of the intracellular signaling kinases ERK1 and p38 and expression of the pro-inflammatory cytokines TNFα and IL-8 [9].

Albumin is an abundant constituent serum protein (35–50 g/l) with a molecular mass of 66.5 kDa. Under normal conditions, albumin cannot penetrate the endothelium and stays in circulation. However, during inflammation, endothelial permeability increases, and albumin exits blood vessels and can be found in damaged tissues.

We hypothesize that the real-time distribution of labeled albumin in the lungs is a feasible and informative method of directly evaluating the abnormal alveolar-capillary permeability that results in lung infiltrates. In this study, we evaluated the feasibility of ^68^Ga-albumin PET/CT to directly visualize the increased alveolar and airway epithelium permeability caused by inflammatory processes in the lungs.

## Results

### Cy7-albumin accumulates in lungs parenchyma after LPS-induced ARDS

**Fig. 1:**
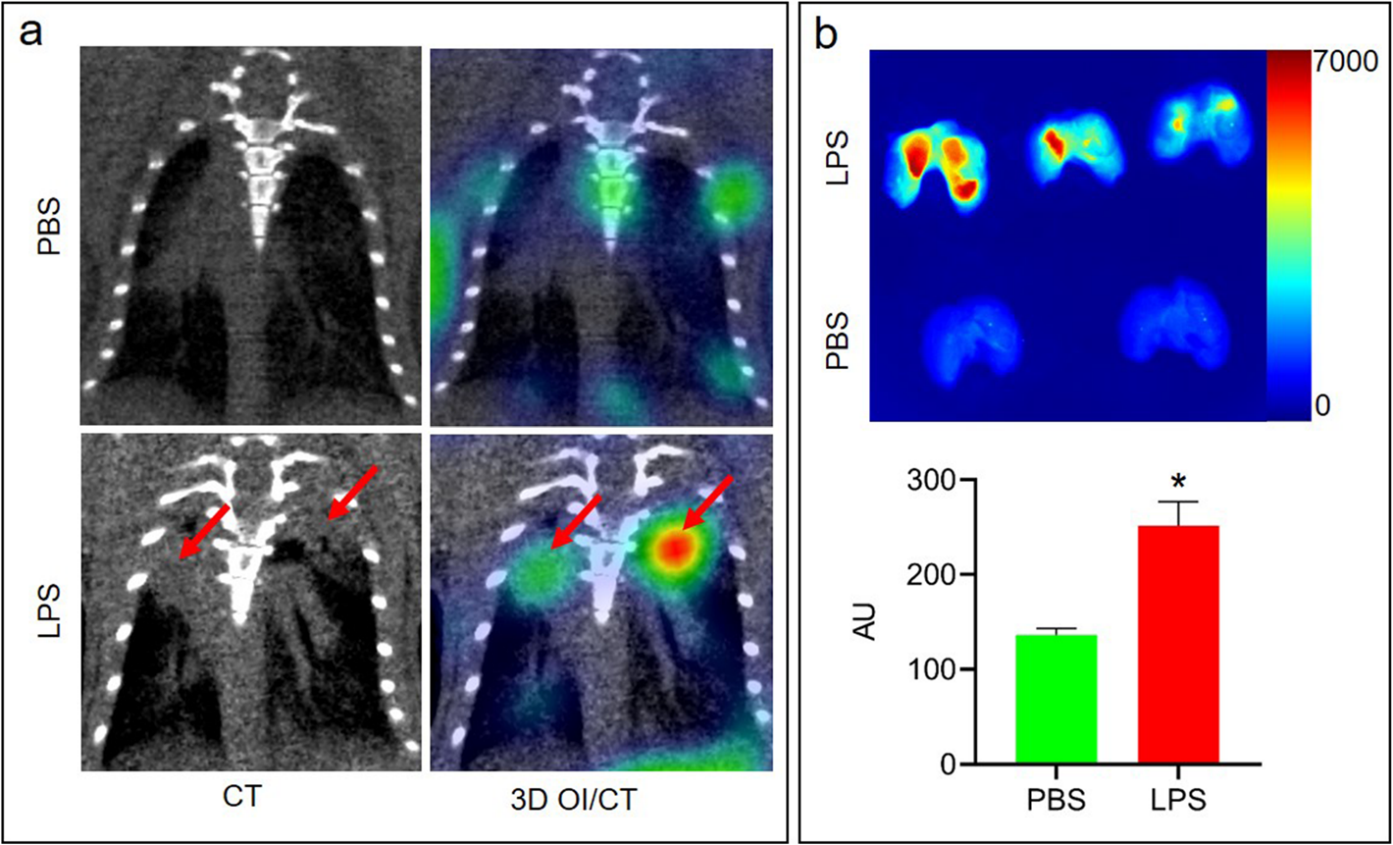
3D OI/CT detection of LPS-induced lung inflammation using Cy7-albumin. **a** representative coronal images of Cy7-albumin uptake in the lungs of the PBS (top, *n* = 4) and LPS (bottom, *n* = 5) treated mice 24 h post injection. CT (left panels) and fusion 3D OI/CT (right panels) are shown. Note that infiltrates visible on CT (red arrows) are co-localized with Cy7-albumin accumulation detected by the 3D optical scan. **b** Cy7-albumin is detected in dissected lungs of mice treated with LPS (*n* = 5) or PBS (*n* = 4, control). *, *p* < 0.01

We hypothesized that systemically injected labeled albumin will leak through the permeabilized alveolar–capillary membrane and allow sensitive real-time visualization of the disease process. To test this hypothesis, we first labeled recombinant mouse albumin with Cy7, a near infrared (NIR) dye, and injected it intravenously into mice 72 h after either intranasal LPS or intranasal PBS (control) treatment. Intranasal LPS treatment induces lung damage followed by formation of protein-rich edema and is thus used as a model of ARDS. Twenty-four hours after Cy7-albumin injection, we imaged mice for Cy7-albumin accumulation in the lungs using 3D optical imaging (OI)/CT. We found Cy7-albumin uptake in the lungs of LPS treated mice (*n* = 5) corresponding to infiltrates on microCT, in contrast to controls (PBS treated, *n* = 4) (Fig. 1a). To confirm the OI/CT results, we euthanized the mice after imaging, and dissected, fixed and imaged the lungs using epi-fluorescent whole lung imaging. Similar to what we observed with OI/CT in live animals, we confirmed substantial (*p* < 0.01) Cy7-albumin accumulation in the lung parenchyma of the LPS-treated lungs (*n* = 5), in contrast to the PBS controls (*n* = 4) (Fig. 1b).

**Fig. 2:**
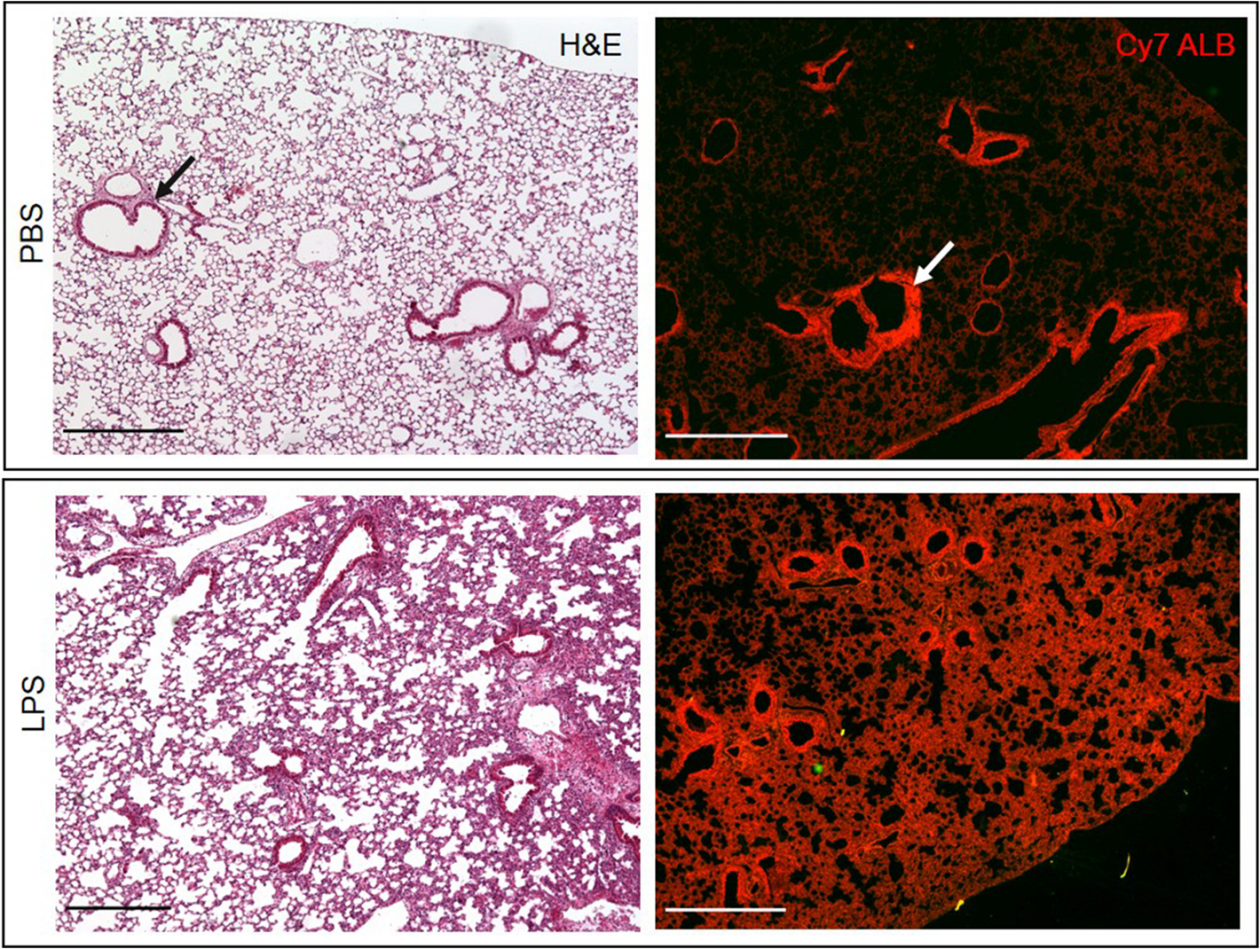
Cy7-albumin uptake in lung parenchyma of mice 72 h after PBS or LPS treatment visualized under a fluorescent microscope.  Bronchovascular bundles (arrows) are visualized on H&E stained sections. Note considerable accumulation of Cy7-albumin (red) in parenchyma of LPS-damaged lungs 24 h post injection. Scale bars, 250 μm

To confirm OI/CT images and demonstrate that the Cy7-albumin correlates with the LPS-induced lung damage, we sectioned the lungs after imaging and compared the Cy7 fluorescence in LPS-treated lungs versus controls (PBS). We found that in LPS-treated mice, there was lung parenchymal accumulation of Cy7-albumin, corresponding to swelling of the parenchyma and hemorrhagic areas seen after H&E staining (Fig. 2). In contrast, in PBS-treated controls, lung morphology was unremarkable and Cy7-albumin was confined to intravascular spaces without significant accumulation in the lung parenchyma (Fig. 2).

### ^68^Ga-albumin does not accumulate in normal lung parenchyma

**Fig. 3:**
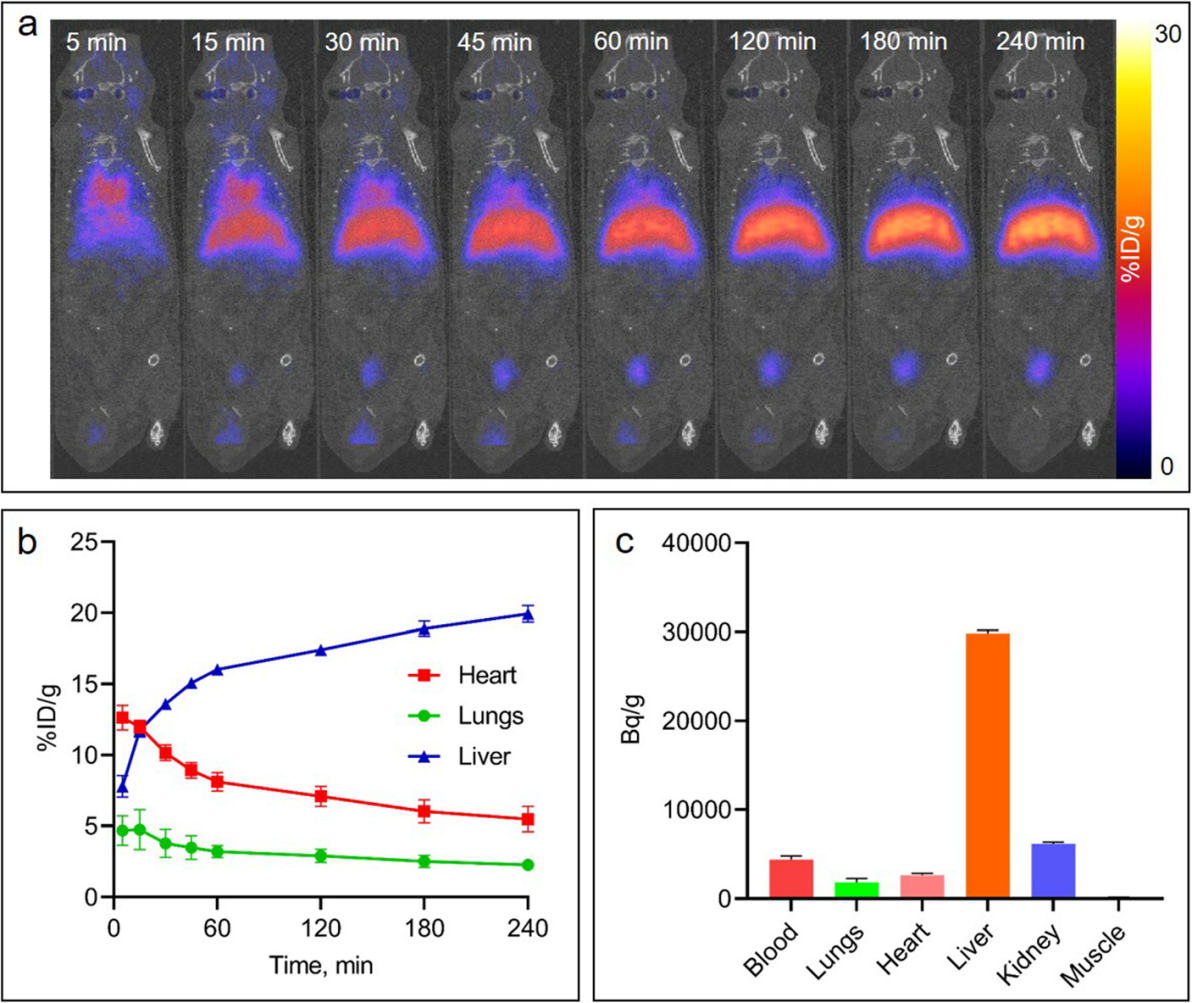
Dynamic 4 h PET imaging after injection of ^68^Ga-albumin. **a** representative coronal images of a 240 min dynamic ^68^Ga-albumin PET scan in an untreated B6 mouse (*n* = 4). **b** dynamic ^68^Ga-albumin activity in the heart, liver and lungs of untreated B6 mice (*n* = 4). **c** biodistribution of ^68^Ga-albumin in tissues of untreated B6 mice (*n* = 4) ~ 4.5 h after injection

To examine if radiolabeled albumin is a feasible translational tracer for imaging real-time alveolar-capillary permeability, we performed whole body dynamic microPET imaging of ^68^Ga-albumin in normal mice to evaluate its physiologic distribution in the lungs and its distribution over an extended period of time (Fig. 3). Mice (*n* = 4) were given a single *iv* dose of ^68^Ga-albumin (~ 7.2 MBq/mouse) and a 4 h dynamic PET scan was acquired (Fig. 3a). We found that ^68^Ga-albumin was retained in blood circulation, including in the heart and large blood vessels, but cleared by at least 50% at 2 h (Fig. 3b). As expected, most of the ^68^Ga-albumin accumulated in the liver (Fig. 3a, b), reaching about 20% ID/g by 240 min. In addition to the liver, we detected some accumulation of ^68^Ga-albumin in the bladder. There was low uptake in peripheral tissues, including in the lung parenchyma of normal mice (Fig. 3a). These results were confirmed post-necropsy by measuring radioactivity in blood and tissues from mice 4 h after ^68^Ga-albumin injection (Fig. 3c).

### ^68^Ga-albumin PET/CT identifies areas of LPS-induced lung permeability

**Fig. 4:**
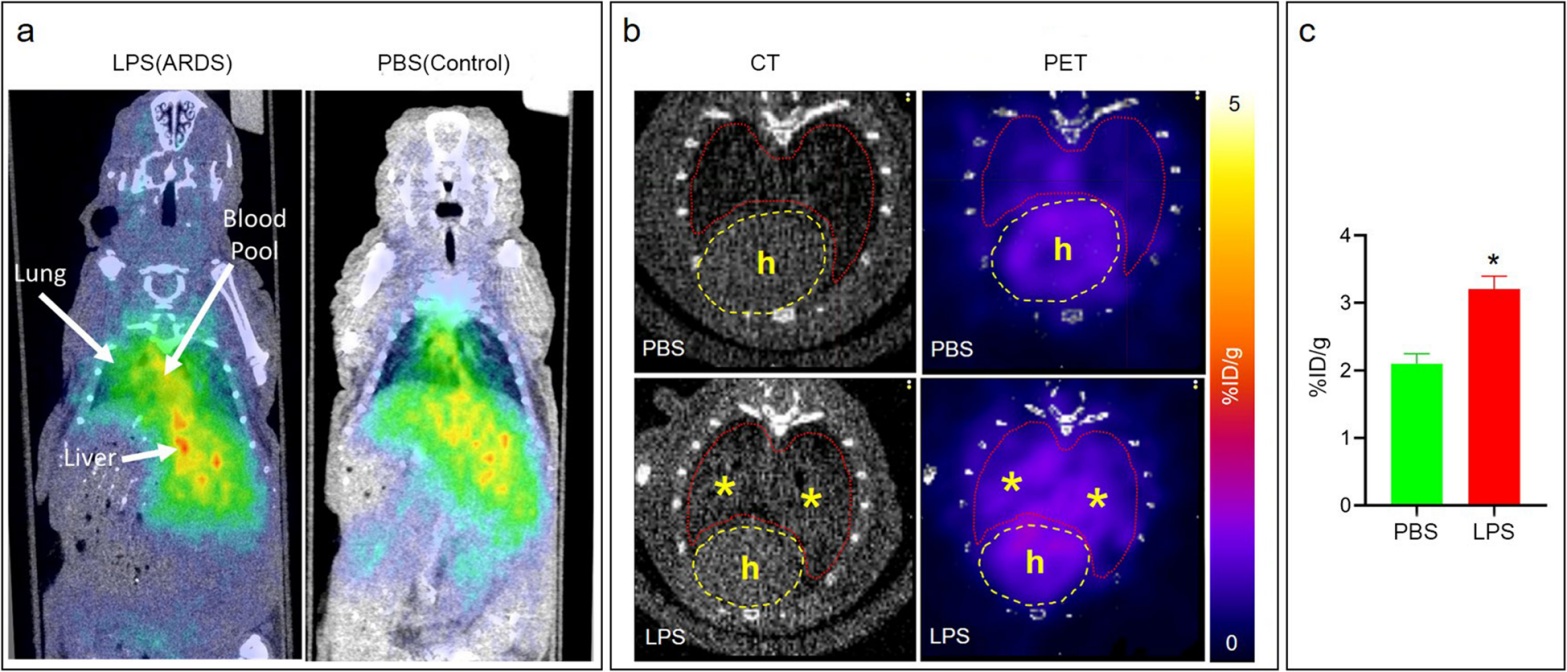
^68^Ga-albumin PET uptake in an LPS model of ARDS. **a** representative whole body coronal ^68^Ga-albumin PET/CT images 4 h after injection demonstrating significantly increased lung activity in LPS treated lungs compared to control, which only demonstrated physiologic uptake in the blood pool (heart) and liver. **b** quantification of ^68^Ga-albumin lung uptake on PET/CT axial images was derived by drawing ROIs on the CT image (*) and calculating the %ID/g from the corresponding PET scan. **c** ^68^Ga-albumin lung uptake calculated from PET/CT images (as shown in **b**). ROIs from lungs were compared between mice treated with intranasal LPS (*n* = 4) and intranasal PBS (*n* = 3), demonstrating significantly higher uptake in lungs of mice treated with LPS. *, *p* < 0.01

Our results using Cy7-albumin demonstrated the potential of labeled albumin as a marker of alveolar-capillary permeability. We therefore tested the lung uptake of ^68^Ga-albumin using PET/CT in mice pre-treated with intranasal LPS versus intranasal PBS. Static 30 min PET scans were acquired 4 h after ^68^Ga-albumin injection followed by microCT for anatomical reference (Fig. 4). We chose 4 h as a balance between blood pool clearance (Fig. 3b) and the 68 min half-life of ^68^Ga. Bq/g: Becquerel / g.

**Fig. 5:**
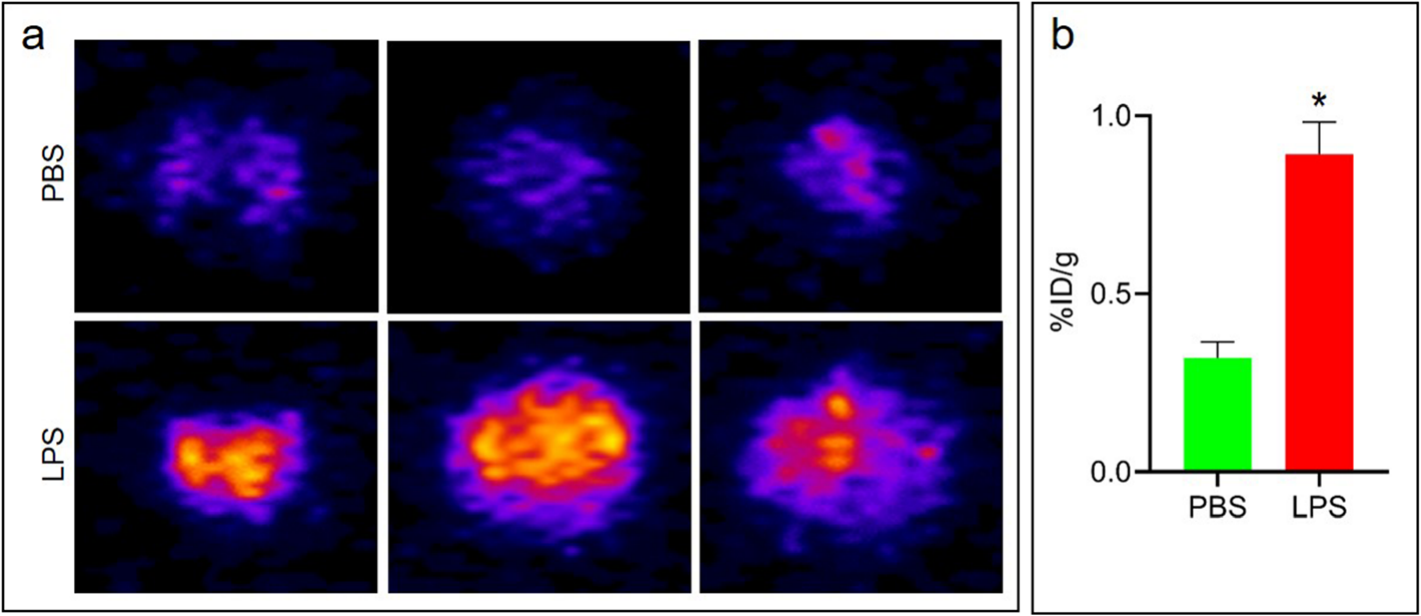
**a** PET scans of dissected lungs from LPS (*n* = 4) and PBS (*n* = 3) treated mice ~ 4.5 h after ^68^Ga-albumin injection. **b** quantification of uptake from (**a**). *p < 0.01

We found significantly (*p* < 0.01) increased ^68^Ga-albumin PET signal in the lungs of LPS treated mice (*n* = 4) corresponding to infiltrates on CT, in contrast to controls (PBS-treated mice, *n* = 3) (Fig. 4). To confirm the PET/CT results, we euthanized the mice immediately after imaging, and imaged just the lungs using PET. Similar to the 3D OI/CT and PET/CT results, we observed significant (*p* < 0.01) ^68^Ga-albumin accumulation in the lungs of the LPS-treated mice, in contrast to the PBS controls (Fig. 5).

## Discussion

We demonstrated accumulation of the both Cy7-albumin and ^68^Ga-albumin in an LPS-lung model of ARDS corresponding to lung parenchymal infiltrates seen on CT. Furthermore, we demonstrate that ^68^Ga-albumin appreciably clears the blood pool after 2 h, which is relevant to evaluating lungs adjacent to the heart and great vessels.

Albumin has long been used as a marker to track vascular integrity. For example, Evan’s blue (EB) dye, which binds serum albumin, is routinely used to test vascular permeability, blood-brain barrier integrity and cardiac function [10–12], but information about barrier integrity is only available during post-mortem analysis. To evaluate our hypothesis, we first labeled albumin with Cy7 and used 3D OI/CT to evaluate its distribution in a mouse model of LPS-induced pneumonitis compared to controls.

LPS is predominantly composed of components of the outer membrane of Gram-negative bacteria and is a potent initiator of inflammation. LPS binds to the CD14/TLR/MD2/LBP receptor complex in monocytes and macrophages to induce production and secretion of pro-inflammatory cytokines [13, 14]. Intranasal LPS administration is an animal model of lung injury displaying many key features of ARDS [1] such as pulmonary edema and leukocyte infiltration, which has become a significant public health concern during the COVID-19 pandemic. We demonstrated that intranasal LPS induced lung infiltrates on CT with corresponding accumulation of Cy7-albumin on 3D OI/CT (Figs. 1 and 2). These results show that 3D OI/CT can be used as a means of real-time imaging of disease induced alveolar-capillary permeability, which can be important in evaluating early treatment effects of new drugs being developed to reduce permeability or treat underlying diseases such as COVID-19-induced pneumonia and ARDS. Using optical imaging to longitudinally visualize real-time pneumonia is a very accessible preclinical modality and can thus play a role in screening compounds for the treatment of COVID-19 related lung disease.

Our promising Cy7-albumin 3D OI/CT results prompted us to see if we can develop a translational PET scan to visualize albumin distribution in diseases that have increased lung permeability, including ARDS, COVID-19 pneumonia, blood-borne sepsis, primary lung infection, gastric aspiration, barotrauma, transfusion associated acute lung injury and hydrostatic chronic heart failure associated pulmonary edema. A previous study in humans already utilized albumin indirectly labeled with ^68^Ga-EB as a blood pool marker to differentiate liver hemangiomas, which showed increased activity, compared to focal hepatic lesions showing decreased activity [15]. Furthermore, the human study demonstrated very low ^68^Ga-EB-albumin uptake in the normal lungs [15], supporting our hypothesis that this might be a good approach to image damaged lungs. While their study demonstrated proof of concept using early imaging with albumin PET as a blood flow marker (30 min after injection), we were interested in imaging the albumin that leaked into the lungs *after* it was sufficiently cleared from the blood pool. We therefore performed whole body microPET imaging of normal mice for an extended period of time (240 min), significantly longer than the 30 min reported when albumin PET was used as a blood flow tracer. Furthermore, we directly labeled albumin using a NOTA bifunctional chelator to minimize any free ^68^Ga from being generated over an extended period of time, as EB displays reversible binding to serum albumin. As expected, ^68^Ga-albumin initially remained in circulation while slowly accumulating in the liver during the first hour, with greater than 50% cleared from circulation at 4 h (Fig. 3). We noted very low uptake of ^68^Ga-albumin in the peripheral tissues of normal mice (Fig. 3). Therefore, to minimize significant blood pool activity in the heart and great vessels that might interfere with adjacent lung, we chose to image at 4 h post injection.

As expected, intranasal LPS injection induced inflammation in the lung parenchyma (Fig. 2) followed by substantial accumulation of the ^68^Ga-albumin in the lungs. This was observed on PET scans of the mice and later confirmed in dissected lungs (Figs. 4 and 5). The differences between the LPS and control mice on PET images are underestimated in mice due to the ROI covering the entire lung, which is only a few mm away from the adjacent background liver and cardiac blood pool activity. The true difference is reflected in Fig. 5, where there is about a 3-fold increase in the LPS treated mice and controls. Due to the close proximity of the lungs, heart, and liver in mice, we could not completely avoid interference in the lower lungs from ^68^Ga liver signal. While liver interference is more significant in mice due to the small size of their lungs, the vast majority of the lung is visible in much larger human lungs, as shown by Zhang et al. [15], although some interference at the lung bases will be inevitable.

Our end translational goal is to use PET imaging to quantitatively evaluate the effect of various experimental therapies on the alveolar-capillary barrier. To accomplish this in real time, it is necessary to use a short half-life radiotracer so we can image pre and post therapy. Furthermore, using ^68^Ga significantly decreases the radiation exposure to the patient due to its comparatively short half-life when juxtaposed with ^64^Cu (T_1/2_ = 12 h) or ^89^Zr (T_1/2_ = 78 h), other radiometals used in nuclear medicine. ^68^Ga is available from a generator in many radiopharmacies that can be easily conjugated using a kit method, which significant eases distribution issues faced by cyclotron-based radiopharmaceuticals such as ^18^F.

In Fig. 5. we demonstrated that at least 50% of the albumin cleared the blood pool at 4 h post injection. This is a very conservative estimate because the ROI was taken to cover the whole lung, including areas adjacent to the liver, which overestimates the actual blood pool retention due to the high liver activity. This overestimation is clearly evident by visualizing the cardiac blood pool in the Fig. 5a, which shows much greater clearance. Thus, we chose to test albumin based imaging due to its established use in humans as well as its ability to stay in the blood pool long enough to accumulate in diseased areas but not too long that it would remain in the blood pool at significant levels at the imaging time of 4 h.

In conclusion, we report here optical or PET imaging of labeled albumin has the potential to detect the real-time lung permeability caused by inflammatory lung diseases or any condition that causes abnormal alveolar-capillary membrane permeability. We are therefore further investigating its use in disease models, such as COVID-19, for early diagnosis of lung disease, predication of disease severity, and for testing novel therapies prior to clinical trials.

## Material and methods

### Animals

C57BL/6NTac mice (B6, Taconic) were maintained on a normal mouse diet. All animal experiments were conducted according to protocols approved by the Institutional Animal Care and Use Committee of Columbia University Medical Center.

### Lipopolysaccharides treatment

Under isoflurane anesthesia, mice were intranasally administered with 1 mg/ml of lipopolysaccharides (LPS) (MilliporeSigma, MA) solution in PBS at dose of 1.5 μl/g. Control animals received equal volumes of intranasal PBS.

### Mouse albumin labeling with Cy7 and ^68^Ga

Mouse serum albumin (ALB) (12–667, MilliporeSigma, MA) was labeled with Sulfo-Cyanine7 NHS ester (Cy7, near infrared die) according to manufacture instructions (Lumiprobe, MD) and purified using a PD10 column (Amersham Biosciences, NJ). Resulting Cy7-albumin (Cy7ALB) concentration was measured using a colorimetric assay and Cy7ALB was stored at -70 °C before use. To label ALB with ^68^Ga, NOTA-NCS (0.42 mg) was added to a 1 ml of 5 mg/ml solution of ALB in saline with 0.1 ml of 0.1 M Na_2_CO_3_ (pH 9.0). The resulting solution was incubated for 30 min at 37 °C using a thermomixer set at 600 rpm. After 30 min, reaction mixture was loaded on preconditioned PD10 column (Amersham Biosciences, NJ) and washed with 1.35 ml of saline. The eluate obtained was discarded and product (NOTA-ALB) was eluted with 2.2 ml saline. ^68^Ga was eluted from a ^68^Ge/^68^Ga generator (Eckert & Ziegler, GalliaPharm, MA). Eluted ^68^Ga (6–8 mCi, 222–296 MBq) was equilibrated to pH 4.0 with 1 M sodium acetate buffer followed by addition of 50 μg of NOTA-ALB. Reaction was incubated at 30 °C for 15 min in a thermomixer (600 rpm). Formation of ^68^Ga-NOTA-ALB was monitored by radio-TLC on ScanRAM 1a (LabLogic, FL) using Varian ITLC-SA strips (Varian, Inc., CA) and 50 mM EDTA (pH 5) as the mobile phase. Specific activity of ^68^Ga-NOTA-ALB was 2850 mCi/mole (43 mCi/g).

### 3D in vivo optical imaging and CT

To detect accumulation of Cy7 albumin in the lungs, control (PBS, *n* = 4) and LPS-treated (*n* = 5) B6 mice were injected *iv* with 75 μl of Cy7 labeled mouse albumin (Cy7ALB) 72 h after LPS/PBS treatment. 3D fluorescent images were acquired under isoflurane anesthesia using a 3D optical/CT scanner (MIlabs, Netherlands) 24 h after Cy7ALB administration. Immediately after 3D optical /CT scan, lungs were dissected, placed in 4% PFA in a 6-well plate, and imaged using the MILabs 3D optical scanner. Images were reconstructed using MILabs OI-PP version 1.8 software.

### PET experiments

72 h after intranasal LPS treatment, B6 mice were injected *iv* with 436–473 μCi (16.1–17.5 MBq) of ^68^Ga-albumin. 4 to 5 h after ^68^Ga-albumin injection, mice were placed into a 4-mouse bed and 30 min static PET images were acquired using micro PET scanner (Siemens, Germany) followed by microCT (MIlabs, Netherlands) on the same bed for anatomical reference. Immediately after PET/CT acquisition, lungs were dissected, placed in 4% PFA in a 6-well culture plate, and a 30 min PET static image of the isolated lungs was acquired. All PET images were reconstructed using 3D-OSEM algorithm with 3-iterations in 256 × 256 matrix (Inveon, Siemens, Germany) and analyzed using VivoQuant ver 4 (Invicro, MA).

### Biodistribution of ^68^Ga-albumin in mice

B6 mice were injected *iv* with a single dose of ^68^Ga-albumin (~ 7.2 MBq/mouse) and 4 h dynamic PET images were acquired using an Inveon microPET scanner (Siemens, Germany) followed by microCT (MILabs, Netherlands) on the same bed for anatomical reference. Immediately after CT, mice were dissected and ^68^Ga-albumin activity in the blood, heart, liver, kidney, and muscle was measured on a Hidex automatic gamma counter (Hidex, Finland).

### Cy7 epifluorescence of the dissected lungs

Lungs were dissected immediately after optical 3D/CT scan and placed in 4% PFA. Cy7 epi fluorescent images of dissected lungs were acquired using MILabs whole-animal optical scanner (MIlabs, Netherlands) using a 20 s exposure time with a 657 excitation / 690 emission.

### Histology

Lungs were fixed overnight in 4% PFA, embedded in paraffin and sectioned. Serial sections were used for H&E staining for morphological references. Cy7 fluorescence images were acquired on Nikon Eclipse Ti (Nikon, Japan).

### Quantification and statistical analysis

Statistical analysis was performed using Prism 8.0. All data are represented as mean ± standard error. Statistical *p*-values were calculated using two-tailed Student’s t-test for unpaired samples.

## Data Availability

Not applicable.

## Abbreviations

ALB: Mouse serum albumin
ALI: Acute lung injury
ARDS: Acute respiratory distress syndrome
CT: Computer tomography
EB: Evan’s blue
LPS: Lipopolysaccharides
NIR: Near infrared fluorescence
OI: Optical imaging
PET: Positron emission tomography
ROI: Region of interest
%ID/g: Percent injected dose per gram
